# A mosaic mutation in the *CLCNKB* gene causing Bartter syndrome: A case report

**DOI:** 10.3389/fped.2023.1034923

**Published:** 2023-04-17

**Authors:** Lan Zhou, Xiaohui Chen, Jiaojiao Xiong, Ling Lei

**Affiliations:** ^1^ Department of Obstetrics and Gynecology, Chongqing Health Center for Women and Children, Chongqing, China; ^2^ Department of Obstetrics and Gynecology, Women and Children's Hospital of Chongqing Medical University, Chongqing, China

**Keywords:** Bartter syndrome, *CLCNKB*, next-generation sequencing, mosaicism, hypokalemia

## Abstract

**Background:**

Type III Bartter syndrome (BS) is an autosomal recessive disease caused by mutations in the *CLCNKB* (chloride voltage-gated channel Kb) gene that encodes CLC-Kb. CLC-Kb is mainly located in the thick ascending limb of Henle's loop and regulates chloride efﬂux from tubular epithelial cells to the interstitium. Type III BS is characterized by metabolic alkalosis, renal salt wasting, hyperreninemia, and hyperaldosteronism with normal blood pressure.

**Case presentation:**

We reported the case of a 3-day-old girl whose initial symptom we diagnosed as jaundice, but we accidentally found metabolic alkalosis. She showed recurrent metabolic alkalosis, hypokalemia, and hypochloremia and also had hyperreninemia and hyperaldosteronism with normal blood pressure. Both oral potassium supplements and potassium infusion therapy were unable to entirely restore the electrolyte imbalance. She was suspected of Bartter syndrome and genetic tests were performed on her and her parents. Next-generation sequencing identified *CLCNKB* gene mutation including heterozygous mutation c.1257delC (p.M421Cfs*58) and a low-level mutation c.595G > T (p.E199*); both mutations were also verified in the parents.

**Conclusion:**

We reported the case of a classic Bartter syndrome in a newborn with a heterozygous frameshift mutation and a mosaic non-sense mutation in the *CLCNKB* gene.

## Introduction

Bartter syndrome (BS) is a rare autosomal recessive inherited disease with clinical characteristics such as metabolic alkalosis, renal salt wasting, hyperreninemia, and hyperaldosteronism with normal blood pressure. Antenatal symptoms may present polyhydramnios and secondary fetal growth retardation (1). BS is categorized into five subtypes according to underlying gene mutations: The *SLC12A1* gene (OMIM: 601678) encoding Na-K-2Cl cotransporter (NKCC2) is responsible for type I BS, the *KCNJ1* gene (OMIM: 241200) encoding renal outer medullary K channel is responsible for type II BS, the *CLCNKB* gene (OMIM: 607364) encoding renal chloride channel-Kb (CLC-Kb) is responsible for type III BS, the *BSND* gene (OMIM: 602522) encoding β-subunit for ClC-Ka and ClC-Kb is responsible for type IVa BS, *CLCNKB* and *CLCNKA* comutation is responsible for type IVb BS, and the *MAGED2* gene (OMIM: 300971) encoding melanoma-associated antigen-D2 (MAGED2) is responsible for type V BS (2).

Classic Bartter syndrome (cBS), also known as type III BS, is thought to have a milder phenotype than prenatal Bartter syndrome (aBS). Renal salt wasting, hypokalemia, metabolic alkalosis, polyuria, polydipsia, and failure to thrive are all symptoms of classic Bartter syndrome, which typically manifests in early childhood. The clinical manifestation of cBS overlaps aBS or Gitelman syndrome, such as hypokalemic metabolic alkalosis and secondary aldosteronism. The onset of classic Bartter syndrome is mostly within 8 months, and late childhood or adulthood onset cases are uncommon (3). *CLCNKB* gene mutation is considered to be the cause of cBS. The *CLCNKB* gene encodes CLC-Kb, which is mainly located in the thick ascending limb of Henle's loop. CLC-Kb functions as a chloride channel; it allows chloride efﬂux from tubular epithelial cells to the interstitium (4).

In this study, we reported a case of a patient with classic BS that carried heterozygous mutation c.1257delC (p.M421Cfs*58) and a mosaic mutation c.595G > T (p.E199*) in the *CLCNKB* gene.

## Case report

### Clinical and biochemical ﬁndings

**Figure 1:**
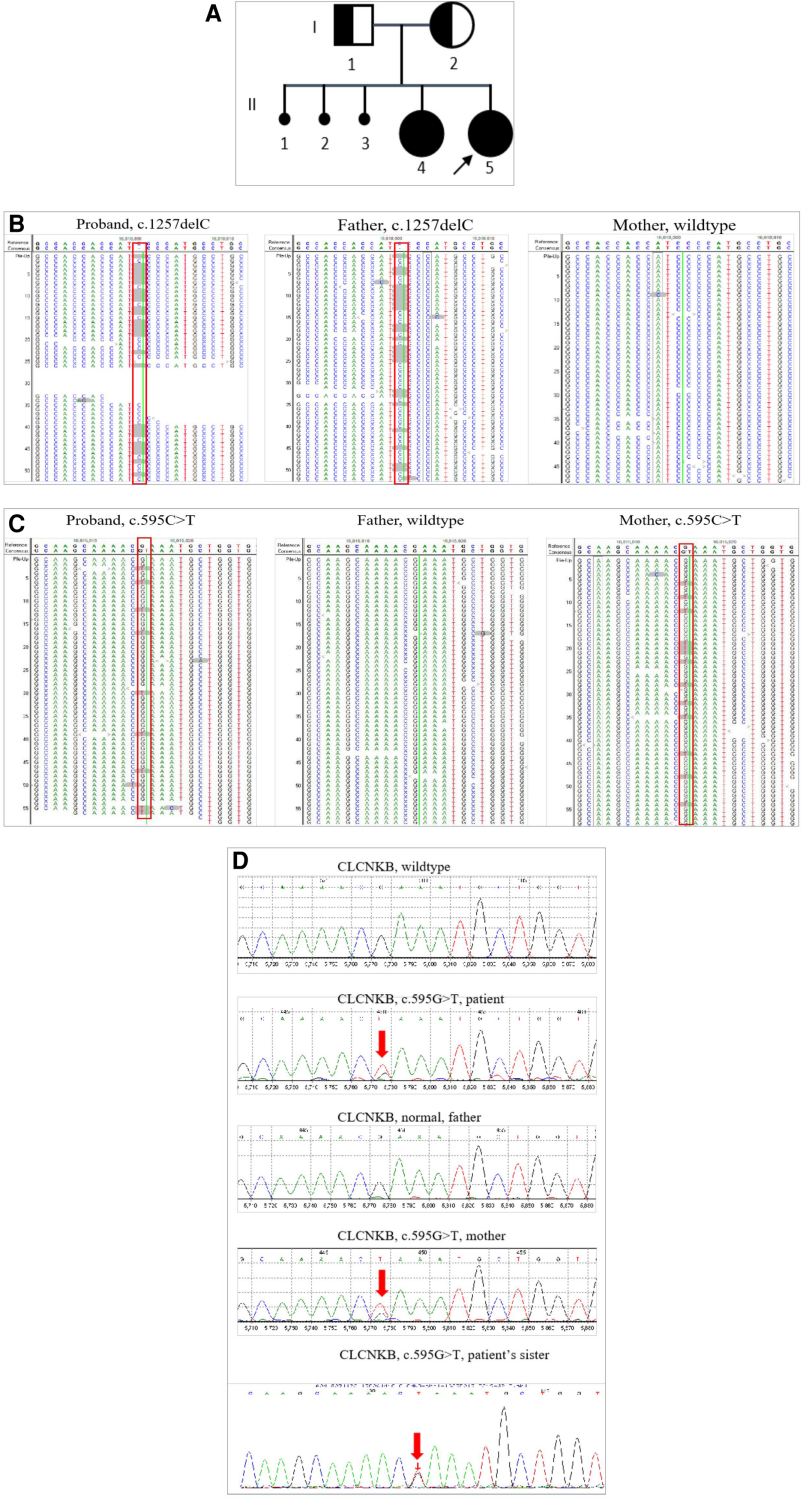
Pedigree of the patient family and Sanger analysis for the mosaic mutation in *CLCKNB*. (**A**) II_2_ indicates the patient, I_1_ and I_2_ indicate the patient's parents; both carried a heterozygous mutation of the *CLCNKB* gene, II_1_ indicates the patient's elder sister with similar clinical symptoms. (**B**) Next-generation sequencing detected c.1257delC in the *CLCNKB* gene in the patient and her parents. (**C**) Next-generation sequencing detected c.595G > T in the *CLCNKB* gene in the patient and her parents. (**D**) Sanger sequencing to confirm the mutation of the *CLCNKB* gene c.595G > T (p.E199*) in the patient’s family.

The girl in this study was the second child in a non-consanguineous Chinese family. Her mother was 27 years old, with gravida 5 para 2. She was born at 37 + 6 gestational weeks. Her birth weight was 2.8 kg and no complications occurred during delivery. Her Apgar scores at 1, 5, and 10 min were (10/10/10), respectively. At 32 gestational weeks of pregnancy, an ultrasound examination revealed a very slight increase in amniotic fluid levels (amniotic fluid index, AFI: 21.9 cm). Her parents were healthy with no family history of disease. The patient's elder sister started showing progressive metabolic alkalosis and hypokalemia at 18 days of life (Figure 1A). The mother's past terminations of pregnancy were not due to medical reasons. On the third day after birth, the patient started to show symptoms of jaundice, and her transcutaneous bilirubinometer reading in the outpatient clinic showed 22.1 mg/mL (mature newborns <12 mg/mL). She was diagnosed with neonatal hyperbilirubinemia (NHB) and admitted to the hospital. After hospitalization, more laboratory tests were conducted: the patient's total bilirubin (TBIL) was 344.9 μmol/L (normal range: 0–23 µmol/L). The patient also revealed an elevated creatine kinase-MB level (CK-MB: 6.2 µg/L) and an elevated high-sensitivity troponin I level (hsTnI: 0.102 µg/L), which indicated possible myocardial damage. An arterial blood gas (ABG) analysis indicated metabolic alkalosis (pH: 7.595, P_O2_: 134 mmHg, P_CO2_: 36.7 mmHg, HCO_3_^−^: 35.6 mmol/L). A blood electrolyte test revealed hypokalemia (K^+^: 2.9 mmol/L), hypochloremia (Cl^−^: 89 mmol/L), and plasma hypoosmolality (mOsm: 271.9). Moreover, the patient showed hyperreninemia (renin: >835.0 µIU/mL), hyperaldosteronism (aldosterone: 1,427.89 pg/mL), and an increased blood Ang II level of 169.84 pg/ml, while her blood pressure was normal (65/44 mmHg) (Table 1). During the physical examination, urine volume was found to be normal, and an ultrasound of the kidneys and adrenal glands showed no sign of nephrolithiasis.

**Table 1:** Laboratory findings of the patient.

Parameter	Result	Reference range
PH	7.595	7.35–7.45
P_O2_	134	80–100 (mmHg)
P_CO2_	36.7	35–45 (mmHg)
Na^+^	133	136–146 (mmol/L)
K^+^	2.9	3.5–4.5 (mmol/L)
Cl^−^	89	98–106 (mmol/L)
Mg^2+^	0.83	0.75–1.2 (mmol/L)
HCO_3_^−^	35.6	22–26 (mmol/L)
mOsm	271.9	280–310
CK-MB	6.2	0–3.1 (µg/L)
hsTnI	0.102	0–0.016 (µg/L)
BP	65/44	SBP:70–90 mmHg^a^
		DBP:50–60 mmHg^a^
Aldosterone	1,421.89	Clinostatism: 29–240 (pg/mL)
		Standing: 31–350 (pg/mL)
Ang II	169.84	Clinostatism:25–129 (pg/mL)
		Standing: 49–252 (pg/mL)
Renin	>835.0	Clinostatism:4–38 (µIU/mL)
		Standing: 4–24 (µIU/mL)

BP, blood pressure; SBP, systolic blood pressure; DBP, diastolic blood pressure; Ang II, Angiotensin II; CK-MB, creatine kinase-MB.

^a^ Blood pressure and its reference range are for the lying position.

A comprehensive timeline of the patient is presented in Table 2.

**Table 2:** Timeline of patient diagnoses and outcomes.

Time	Diagnosis	Measures	Outcome
3 days	NHB	Blue light	Improved
7 days	Metabolic alkalosis, hypokalemia, hypochloremia	Potassium supplement	Recurrent
9 days	Suspected Bartter syndrome	NGS for candidate genes	*CLCNKB* variants
Long-term	Bartter syndrome	Potassium supplement, indomethacin	Persistent hypokalemia, hypomagnesemia

### Genetic analysis and results

Peripheral blood samples of the patient and her parents were used for genetic analysis. We used custom-designed NimbleGen SeqCap probes (Roche NimbleGen, Madison, WI, United States) for in-solution hybridization to amply target sequences. Clinical exon sequencing (CES) candidate genes include more than 5,000 disease-causing genes selected from reports in the OMIM, HGMD, and reliable literature, and known pathogenic variants in deep introns and non-coding regions in targeted genes were also included (Supplementary Table). The DNA samples were indexed and sequenced by using the Illumina sequencer (San Diego, CA, United States). The average coverage depth was about 200 × with over 98% of the target regions covered by at least 20 reads. Nucleotide changes found in the aligned reads were pulled and analyzed using the NextGENe software (Version 2.4.2) (SoftGenetics, State College, PA, United States). Sequence variants were annotated and included population (gnomAD, 1,000 Genomes, dbSNP) and variant databases (Clinvar, HGMD). Online software Polyphen-2 and SIFT were used for *in silico* analysis of missense variants. The variants were classified as “Pathogenic,” “Likely Pathogenic,” “Uncertain Significance,” “Likely Benign,” or “Benign” according to the American College of Medical Genetics and Genomics (ACMG) guidelines. A trio-clinical exome sequencing (AmCare Genomics Lab, Guangzhou) was performed on the patient and her parents. A frameshift mutation c.1257delC (p.M421Cfs*58) in exon 13 of the *CLCNKB* gene was found in the patient, which was inherited from the patient's asymptomatic father (Figure 1A). Other candidate genes of Bartter syndrome and Gitelman syndrome such as *SLC12A1*, *KCNJ1*, *BSND*, *CLCNKA*, *MAGED2*, and *SLC12A3* did not reveal any significant variants. Bartter syndrome is an autosomal recessive disease, in which both alleles of the *CLCNKB* gene are affected by recessive mutations to present symptoms. However, CES detected no variants in the *trans* allele of the *CLCNKB* gene at first. Given that both the patient and her elder sister presented typical cBS clinical characteristics, we reviewed the data origin and manually searched for a non-sense mutation c.595G > T (p.E199*) in exon 6 of the *CLCNKB* gene with a low-variant allele frequency (VAF) of 16.44%. This mutation was also verified in the patient's mother with a VAF of 20.8% (Figure 1B,C). Therefore, we speculated that mutation c.595G > T (p.E199*) in the *CLCNKB* gene was a low-grade mosaic mutation. Sanger sequencing was used to confirm the low VAF mutation (Figure 1D). Therefore, the diagnosis of Bartter syndrome was confirmed.

### Treatment and follow-up

The patient was treated with blue light for NHB and potassium infusion therapy to reverse the electrolyte disturbance. After 7 days of blue light treatment, hyperbilirubinemia significantly reduced, and blood TBIL dropped to 94 µmol/L. The patient was followed up for 22 months. She accepted daily oral potassium chloride supplementation and indomethacin (7.5 mg, tid). On the last follow-up, the patient's metabolic alkalosis had significantly improved, but hypokalemia (3.07 mmol/L) and hypomagnesemia (0.46 mmol/L) were still present. No obvious developmental delay or a degeneration of the renal function was observed in the patient.

## Discussion

**Figure 2:**
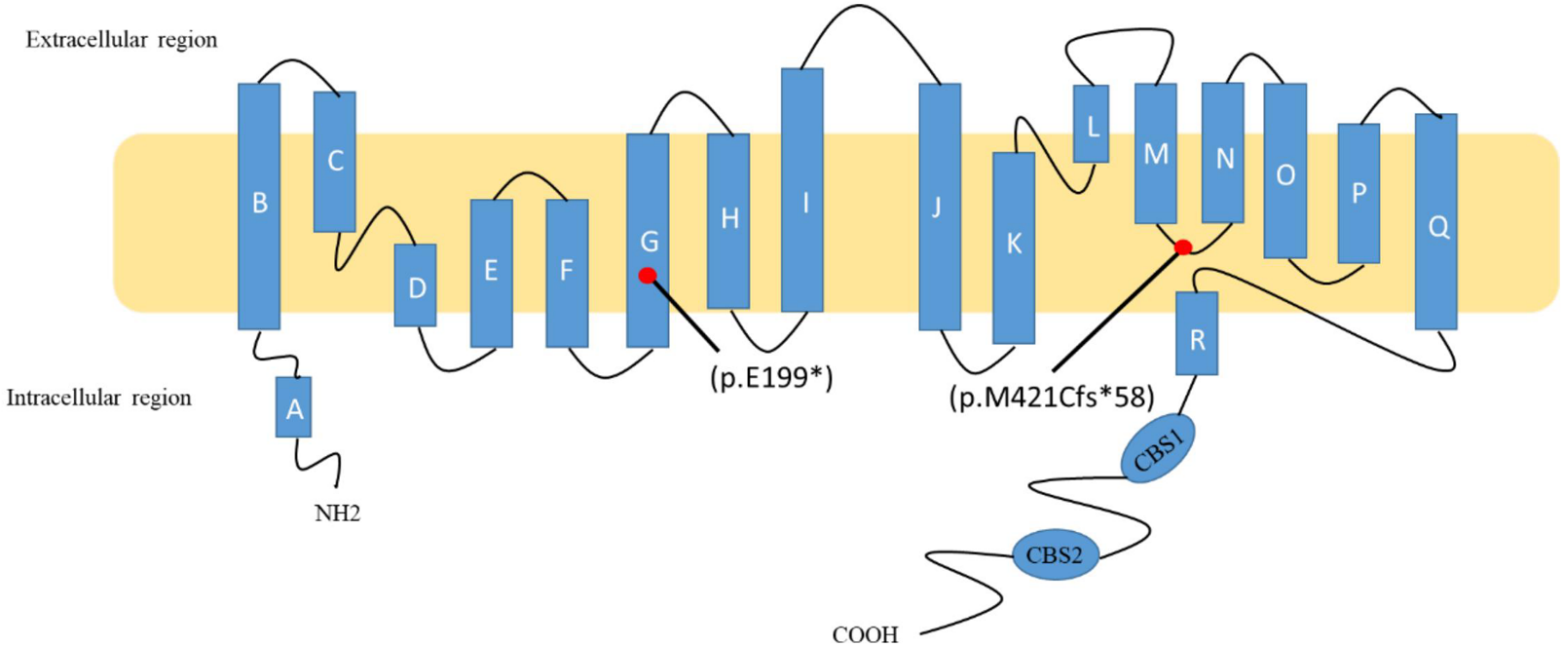
Schematic diagram of ClC-Kb and localization of two mutations. The c.1257delC (p.M421Cfs*58) mutation is located in the linker between helices M and N, while the c.595G > T (p.E199*) mutation is found in the middle of helices G.

CLC-Kb, a member of the CLC chloride channel family, is encoded by the *CLCNKB* gene. CLC-Kb controls chloride reabsorption by residing in the thick ascending limb of Henle's loop, distal convoluted tubules, and cortical collecting tubules (5). CLC-Kb comprises 687 amino acids and forms homodimers at the plasma membrane, with each monomer defined by 18 α-helices (from A to R) spanning the membrane by an antiparallel structure. The carboxy terminus of CLC-Kb lands in the intracellular region with two cystathionine-β-synthase domains (6). The c.1257delC (p.M421Cfs*58) mutation is located in the linker between helices M and N, while the c.595G > T (p.E199*) mutation is found in the middle of helices G. The CLC-Kb structure is shown according to what is prescribed by Jentsch et al. (7) (Figure 2).

In our study, despite the patient's initial symptom of jaundice and the absence of other obvious symptoms prior to admission, her ABG analysis revealed metabolic alkalosis and electrolyte disturbance. Physiologic jaundice is common in infants, but as the patient exhibits persistent metabolic alkalosis even after her jaundice relieved, we considered the jaundice and metabolic disturbance as independent events. The patient represented typical type III Bartter syndrome, including hyperaldosteronism, hyperreninemia, and recurrent metabolic alkalosis, along with normal blood pressure. Increased CK-MB levels could be explained by an elevated aldosteronism and hypokalemia (8). Since the clinical symptoms of type III Bartter syndrome are similar to those of aBS or Gitelman syndrome, accurate diagnosis assumes importance. Gitelman syndrome mostly features hypomagnesemia and hypercalciuria, whereas antenatal Bartter syndrome typically displays severe polyhydramnios with nephrocalcinosis (9).. In this patient case, only in her mother was there was a slight increase in the amniotic fluid levels in the third trimester, which failed to reach the diagnosis of polyhydramnios, and the patient showed no signs of nephrocalcinosis. The phenotypes of type III Bartter syndrome can be highly variable, and we used next-generation sequencing (NGS) to uncover the disease-causing gene to confirm the diagnosis.

Mutation c.1257delC (p.M421Cfs*58) in the *CLCNKB* gene has been identified by Han et al. in a Chinese patient (10). This mutation is associated with a single cytosine deletion at nucleotide 1,257, resulting in a frameshift mutation from methionine to cysteine at position 421 in the protein and the protein is truncated at position 478. This mutation was classified as “Pathogenic” by the 2015 ACMG (11). Mutation c.595G > T (p.E199*) in the *CLCNKB* gene has been reported by Lee et al. in Gitelman-like syndrome (12). A cytosine to thymine occurred at nucleotide 595, resulting in a single amino acid non-sense mutation from glutamic to stop codon at position 199, causing the protein to truncate. This mutation was classified as “Pathogenic” by the 2015 ACMG. Han et al. (10) conducted genotype and phenotype association analysis for CLCNKB mutations. They found that null variants (CL mutation) were associated with a significantly early age of onset and worse alkalosis. However, they did not describe in detail the phenotypes of the patient with these two compound heterozygous mutations. Our patient case showed that CL/CL mutations presented an early onset (3 days of life) and recurrent alkalosis. The VAF of mutation c.595G > T (p.E199*) in the patient and her mother were 16.44% and 20.8%, respectively. At first, we considered it to be somatic mosaicism. There was only one report that mentioned trisomy 3 mosaicism in a patient with Bartter syndrome, but the relationship between these two events was unknown (13). In addition to this report, there was no previous study about somatic mosaicism in *CLCNKB* resulting in Bartter syndrome. The term “mosaicism” means two or more cell populations with different genotypes when an organism develops. Mosaicism can be inherited by offspring if it occurs in the germline (14). However, in our case, a kidney biopsy of the patient or her mother's gonadal tissue was unavailable, and therefore, we were unable to confirm the exact VAF in the lesion. In genetic diseases, mosaicism is a common but easily missed condition during diagnosis. The difficulty in diagnosing mosaicism does not lie in technological limitations but in locating where the variants occur. The phenotypes caused by mosaicism can be heterogeneous, mainly depending on the specific tissues that the variants expressed. Today, next-generation sequencing is the most efficient way to detect single nucleotide variants and small insertions and deletions, and high-depth sequencing allows the detection of VAF even up to 1% (15). Yet, VAF mutations below 20% in NGS outcomes tend to be easily neglected because they were typically deemed background signals or low-quality mutations and are being filtered now.

This patient case highlighted the importance of detecting mosaic mutation in clinical practice. A comprehensive family history of patients should be acquired, especially in those who exhibit symptoms highly similar to genetic disorders. These mutations should be taken into consideration, especially in patients who exhibit clear clinical signs. Clinical-exome sequencing or whole-exome sequencing is the most comprehensive analysis to accurately identify potential mosaic patients.

## Conclusion

In conclusion, recurrent metabolic alkalosis, hyperreninemia, and hyperaldosteronism with normal blood pressure should arouse the clinician’s suspicion of Bartter syndrome. A genetic test plays an important role in the confirmation of diagnosis, especially in different types of Bartter syndrome and Gitelman syndrome. NGS revealed a heterozygous frameshift mutation and a low VAF non-sense mutation in the *CLCNKB* gene. This case shows the importance of genetic analysis in BS diagnosis.

## Data Availability

The data in this article are not publicly available because of issues involving the patient’s anonymity. Requests to access the data should be directed to the corresponding author.
